# Diagnostic accuracy of *Schistoso* *ma* *ICT Ig* *G* *-* *IgM* and comparison to other used techniques screening urinary schistosomiasis in Nigeria

**DOI:** 10.1515/almed-2020-0093

**Published:** 2021-02-09

**Authors:** Robert Soumay Houmsou, Binga Emmanuel Wama, Hemen Agere, John Ador Uniga, Timothy Jerry Jerry, Paul Azuaga, Elizabeth Une Amuta, Santaya Larit Kela

**Affiliations:** Department of Biological Sciences, Taraba State University, Jalingo, Nigeria; Department of Biological Sciences, Federal University Wukari, Wukari, Taraba State, Nigeria; Department of Paediatric Unit, Federal Medical Centre, Jalingo, Taraba State, Nigeria; Department of Zoology, Federal University of Agriculture, Makurdi, Benue State, Nigeria; Department of Biological Sciences, Federal University Kashere, Gombe, Gombe State, Nigeria

**Keywords:** comparison, evaluation, Nigeria, schistosomiasis, urinary

## Abstract

**Objectives:**

Schistosomiasis is a blood fluke parasitic illness affecting human lives in rural endemic areas. This study evaluated the performance of *Schistosoma* *ICT Ig* *G* *-* *IgM* for screening urinary schistosomiasis in Nigeria.

**Methods:**

Three hundred and seventy four (374) urine samples were examined. Reagent strips, urine filtration and *Schistosoma* *ICT Ig* *G* *-* *IgM* were used for analysis. *Schistosoma* *ICT Ig* *G* *-* *IgM* used 2 mL of each serum for serological examination. Then, 3 mL of each preserved serum was sent to LDBIO Diagnostics, France for re-examination with *Schistosoma* ICT IgG-IgM and confirmation with SCHISTO Western blot (WB) IgG. The performance of the index tests was determined using sensitivity (Se), specificity (Sp), positive predictive value (PPV), negative predictive value (NPV) and positive likelihood ratio (PLR). The Youden index (YI) and diagnostic accuracy (DA) were used to determine the accuracy of each test. The statistical significance was at p-value ≤0.05.

**Results:**

The test had a sensitivity of 94.9%, specificity of 63.9%, positive predictive value of 72.4%, negative predictive value of 92.6%, and positive likelihood ratio of 2.62. *Schistosoma ICT Ig* *G* *-* *IgM* had a good Cohen’s kappa index (κ=0.68), good Youden index (YI=0.58) and good diagnostic accuracy (DA=0.78).

**Conclusions:**

*Schistosoma* *ICT Ig* *G* *-* *IgM* has proven to be the best technique for the screening of urinary schistosomiasis in Nigeria.

## Introduction

Schistosomiasis is a neglected tropical disease caused by *Schistosoma* sp which are trematode worms. The disease is an acute and chronic illness affecting humans living in rural areas. It has been estimated that 240 million people are infected around the world and about 90% of them are found in sub-Saharan Africa [1]. In sub-Saharan Africa, schistosomiasis is a serious public health for rural inhabitants. They are usually exposed to infection through their agricultural practices of farming rice, recreational and domestic activities, fishing, and swimming in infested ponds or rivers [2], [3]. In Nigeria, urinary schistosomiasis affects rural inhabitants. It varied respectively from various locations, 62.0% in Kwara State and 45.6% in Kano State [4], [5]. *Schistosoma mansoni* was also found among rural people but with low infection in different communities [6], [7].

The usual laboratory examination of schistosomiasis in health centers of rural and urban areas was based on reagent strips [8], [9]. Urine filtration and egg visualization used to be the technique for diagnosing urinary schistosomiasis in Africa. The test was unsatisfactory because it had a limitation in single diagnosis of infected individuals and was also labor intensive [10].

*Schistosoma* *ICT Ig* *G* *-* *IgM* was developed recently as a rapid and qualitative test. This study evaluated the first screening performance of *Schistosoma* *ICT Ig* *G* *-* *IgM* in Nigeria 6 months after its commercial availability. This research compared *Schistosoma* *ICT Ig* *G* *-* *IgM* to reagent strips and filtration usually used on the field.

## Materials and methods

### Study area

The study was conducted in Takum Local Government Area (LGA) located at latitude 07° 15′N and longitude 09° 59′E of Taraba State, Northeast, Nigeria. It is bordered to the South by the Republic of Cameroon, to the West by Ussa LGA, to the North by Donga LGA and to the East by Katsina-Ala LGA, Benue State. The Local Government Area is sub-divided into 10 wards among which seven wards were selected. The selection was based on the presence of functional Primary Health Care Centers, where inhabitants were referred to for treatment. The areas were all drained by substantial rivers from which inhabitants depend on for their daily home activities. The wards selected were Barki-Lissa, Birama, Chanchanji, Kashimbila, Manya, Malumshe, and Sufa (Supplementary Figure 1).

The climate of the area is tropical with vegetation characterized by a typical guinea savannah interspersed with gallery forest. There are two distinct seasons: the rainy season, which starts from mid-March to mid-October and the dry season from mid-October to mid-March. The annual rainfall ranges between 1,200 – 2,000 mm. The average temperature is between 24 and 32 °C with a peak at 37 °C in March and April.

The area is inhabited by civil servants, agricultural business people and peasant farmers. The Local Government is an area of intense agricultural and fishing activities.

### Study design

The research was a prospective study for the use of *Schistosoma ICT IgG-IgM* on the field. Urine filtration, microhaematuria and proteinuria were used as index tests. Serological samples were sent to LDBIO Diagnostics, Lyon, France for re-examination with *Schistosoma ICT IgG-IgM* and confirmation with SCHISTO II Western blot (WB) IgG.

### Ethical permission

Prior to the commencement of the study, ethical permission was obtained from the Directorate of Health, Takum LGA (TLG/PHC/403) and shown to the Ethical Committee of Federal Medical Center, Jalingo, Taraba State.

### Eligibility of participants

The local chief of each community ward was contacted and briefed about the significance of the study three days ahead of sampling. The oral expression with the participants to collect their urine and blood for examination was in “*jukun*”, a local language in the area. The inhabitants of each ward were informed to gather at their local chief premise for interaction a day before collection of urine and stool samples. Each local inhabitant was informed to convene respectively to his functional Primary Health Care ward for collection of samples. Adults, children and their parents or guardians to be enrolled were verbally interviewed in their tribal language ‘*jukun*’. Participants conveniently followed the instructions before sampling.

### Sample size, exclusion and inclusion criteria

The sample size of the subjects was calculated by the formula:

$$N=\frac{Z^{2}.P\left(1-P\right).D}{E^{2}}$$

where, *P*=50% is the estimated prevalence of urinary schistosomiasis. *D*=1, reflects the design effect of the simple random sampling used for enrollment of subjects. *E* ^2^=0.0025, precision (margin error, which is 10% of the estimated prevalence). *Z*=1.96, for two tails.

Four hundred and twenty (420) individuals were enrolled for the study. Sixty (60) individuals were randomly sampled in each of the seven wards. Participants that were ill and had urinary illness within 2 weeks were excluded. Women on menstruation were also excluded from the study. Each subject who refused to give 5 mL of blood to prepare plasma was equally excluded. Subjects that also refused to give 10 mL of urine for filtration technique were disengaged. Some individuals refused to participate because of their cultural beliefs on the use of their blood and urine. Participants that fulfilled all the criteria to give their blood and urine samples were included in the study. Participants that were diagnosed of urinary schistosomiasis were treated immediately with Praziquantel.

### Laboratory examinations

Subjects and parents of the children were informed on different examination methods. The urine filtration and reagent strips were used for urine samples while Kato-Katz (Sterlitech Corporation, Kent, USA) was used for faecal examination. *Schistosoma ICT IgG-IgM* was used for sera. Anonymous and coded duplicates of sera used on the field were sent to LDBIO Diagnostics, Lyon, France for re-examination with *Schistosoma ICT IgG-IgM.* SCHISTO II WB IgG was used for confirmatory diagnostic decision of sera collected.

### Field determination of microhaematuria and proteinuria

Reagent strips (Medi-Test Combi 9, MACHERY-NAGEL, Germany) were used for determination of microhaematuria (Ery/μL) and proteinuria (mg/dL). Microhaematuria was determined as: 0=negative, Ca.10=+, Ca.50=++ and Ca.100=+++. Proteinuria was measured as: 0=negative, Ca.30=+, Ca.100=++ and Ca.500=+++

### Filtration and Kato-Katz techniques

Filtration is a field technique for urinary schistosomiasis. A 10 mL syringe, swinney polypropylene filter holder (13 mm diameter) and polycarbonate membrane filters (12.0 μm porosity) (Sterlitech Corporation, Kent, USA) were used during urine filtration. Urine samples were reported as: 0 eggs/10 mL of urine (not infected), 1–49 eggs/10 mL of urine (lightly infected) and >50 eggs/10 mL of urine (heavily infected) using WHO standard [11].

Faecal sample examination was conducted using the Kato-Katz and faecal concentration techniques. The infection was classified as <100 epg (low parasitic load), 100–399 epg (medium parasitic load) and >400 epg (high parasitic load) [11].

### *Schistosoma ICT Ig* *G*-*IgM*

*Schistosoma ICT Ig* *G* *-* *IgM* is a newly developed rapid and qualitative test. It is based on immune-chromatography technology detecting simultaneously IgG and IgM. A 10 mL of blood sample was collected from each subject. Then 5 mL of serum was prepared from each blood sample. Each *Schistosoma ICT Ig* *G* *-* *IgM* used 30 μL of each prepared serum for schistosomiasis examination. The cassette assay determined the positive, negative and invalid tests between 20 and 30 min. The test procedure was described as follows: 30 μL of serum and an eluent were firstly dispensed in the sample well of each assay. The test became positive when a red test band “T” appeared. It was rather negative when a control blue band “C” appeared. The test was invalid when there was no appearance of “T” or “C” bands. The details for use of *Schistosoma ICT Ig* *G* *-* *IgM* are found in the instructions [12].

The remaining 3 mL were preserved in 5 mL eppendorf tubes. Thirty microliters (30 μL) of sodium azide (NaN_3_) at concentration of 0.02% (w/v) were added on each serum. In the laboratory, each serum was preserved for 3 days in a freezer at −4 °C. They were sent to LDBIO Diagnostics, Lyon, France for re-examination with *Schistosoma ICT IgG-IgM.* SCHISTO II WB IgG was used for confirmation [12]. The results of *Schistosoma ICT IgG-IgM* on the field were considered for this study as re-examination results gave a minimal difference.

### SCHISTO II Western blot (WB) IgG

SCHISTO II WB IgG is a qualitative WB technique. It is based on a serologic IgG diagnosis by immunoblot assay. It was used to confirm the positive or equivocal result from each *Schistosoma ICT IgG-IgM.* The procedure used the acrylamide gels (13%) and the discontinuous sodium dodecyl sulfate buffer which electrophoresed the *Schistosoma* *mansoni* and *Schistosoma haematobium* antigens solution [13]. The molecular weight standard used a mixture of biotinylated and prestained proteins (myosin, β-galactosidase, phosphorylase *b*, bovine serum albumin, ovalbumin, carbonic anhydrase, trypsin inhibitor, lysozyme, and aprotinin). The gels moved faster than the 30-kDa trypsin inhibitor (which had been prestained blue) that reached the bottoms of the gels. The proteins were then transferred to nitrocellulose sheets with slight modifications which were also coated with Tris-NaCl (pH 7.4) containing 5% non-fat milk [14]. Tris-NaCl washed the blots twice, dried and separated it into 4 mm wide strips. The antigen strips were incubated with diluted sera (1:50 in Tris-NaCl) sample buffer for 90 min. The strips were incubated with an anti-human immunoglobulin G-alkaline phosphatase conjugate for 60 min after a washing step with Tris-NaCl washing buffer. Then, the strips were washed again. The sera recognized the protein fractions and were revealed by the corresponding substrate-chromogenic solution that contained nitroblue tetrazolium and 5-bromo-4-chloro-3-indolyl-phosphate. The strips were washed with distilled water and the reaction stopped. The strips were dried and fixed on paper for reading and storage. Positive and negative control results were tested in each assay.

### Data analysis

Microsoft Excel was used to collate and sort the entire data. XLSTAT 2017 evaluated the sensitivity (Se), specificity (Sp), negative predictive value (NPV), positive predictive value (PPV) and positive likelihood ratio (PLR) of the tests. The Youden index (YI) for each test was calculated as sensitivity + specificity – 1. The diagnostic accuracy (DA) was calculated as DA=True Positive + True Negative/True Positive + True Negative + False Positive + False Negative. IBM SPSS 23.0 evaluated the Cohen’s kappa measurement and concordance of the index tests. The Cohen’s Kappa index (κ) values were interpreted as follows: <0.20 (poor agreement); 0.20 – 0.40 (fair agreement); 0.41 – 0.60 (moderate agreement); 0.61 – 0.80 (good agreement) and 0.81 – 1.00 (very good agreement). The confidence interval (CI) 95% and p-value≤ 0.05 were used as statistical significance of the tests.

## Results

### Flow chart of participants

The age of participants was from 3 to 51 years. The mean age ± standard deviation (SD) was 12.8 ± 10.7. Participants were males (46.2%) and females (53.7%).

**Figure 1:**
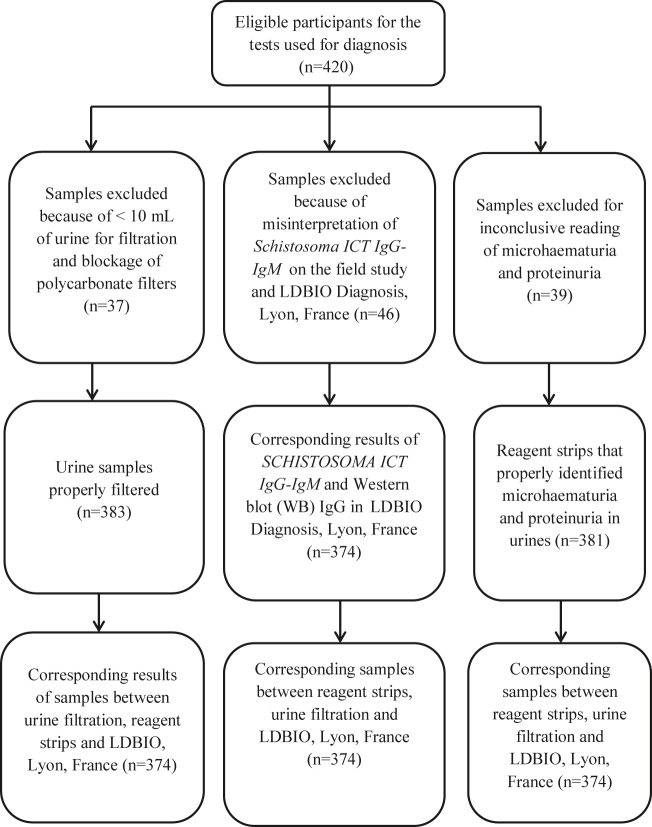
Study flow chart of inclusion and exclusion of participants.

Figure 1 shows the flow chart of participants in the study. Participants were instructed on the sampling criteria. Then, the study randomly selected 60 individuals from each ward. Eligible participants for the study were 420 individuals. Fourty six (n=46) of *Schistosoma ICT Ig* *G* *-* *IgM* tests were not accepted because of misinterpretation between the laboratory technicians. Thirty seven (n=37) urine samples were not used because of <10 mL of urine for filtration (n=18) and non-filtration of polycarbonate filters (n=19). Some reagent strips had inconclusive reading of proteinuria (n=21) and microhaematuria (n=18).

At the end, 374 samples had corresponding results between reagent strips, filtration, *Schistosoma* *ICT IgG-IgM* and SCHISTO II WB IgG.

### Faecal examination

The faecal samples examined had no infection with *S. mansoni*. The study reported intestinal parasites and were not included in this study. They were *Trichuris trichiura* (12.2%), *Ascaris lumbricoides* (8.2%), *Enterobius vermicularis* (6.1%), *Taenia* sp (4.8%) and *Strongyloides stercoralis* (3.2%)*.*

### Concordance of positive results between the index tests (microhaematuria, proteinuria, filtration and *Schistosoma ICT IgG-IgM*) and standard WB IgG technique

Table 1 shows the concordance of the index tests with the standard WB IgG. Microhaematuria had the lowest positivity (6.1%) while *Schistosoma ICT IgG-IgM* had the highest (44.9%). The concordance result of *Schistosoma ICT IgG-IgM* with the standard WB IgG immunoblot assay had the highest Cohen’s kappa (κ*=*0.68). Filtration results did not have a good Cohen’s kappa (κ*=*0.04) and did not also concord with the WB IgG.

**Table 1:** Concordance of positive results between the index tests (microhaematuria, proteinuria, filtration and *Schistosoma ICT IgG-IgM*) and standard Western blot (WB) IgG technique.

WB IgG					
	Negative	Positive	Total	Cohen’s	
				kappa, κ	[95% CI]
Microhaematuria				0.05	0.02 – 0.08
Negative, n (%)	181 (48.4)	154 (41.2)	335 (89.6)		
Positive, n (%)	16 (4.3)	23 (6.1)	39 (10.4)		
Total, n (%)	197 (52.7)	177 (47.3)	374		
Proteinuria				0.13	0.07 – 0.32
Negative, n (%)	117 (31.3)	81 (21.7)	198 (52.9)		
Positive, n (%)	80 (21.4)	96 (25.7)	176 (47.1)		
Total, n (%)	197 (52.7)	177 (47.3)	374		
Filtration				0.04	0.01 – 0.07
Negative, n (%)	136 (36.4)	114 (30.5)	250 (66.8)		
Positive, n (%)	61 (16.3)	63 (16.8)	124 (33.2)		
Total, n (%)	197 (52.7)	177 (47.3)	374		
*Schistosoma ICT IgG-IgM*				0.68	0.41 – 0.83
Negative, n (%)	126 (33.7)	9 ( 2.4)	135 (36.1)		
Positive, n (%)	71 (19.0)	168 (44.9)	239 (63.9)		
Total, n (%)	197 (52.7)	177 (47.3)	374		

Secondary information: κ≤0, no agreement; κ=0–0.20, poor agreement; κ=0.21–0.40, fair agreement; κ=0.41–0.60, moderate agreement; κ=0.61–0.80, good agreement; κ=0.81–1.00, very good agreement.

### Diagnostic performance of tests used for screening urinary schistosomiasis in rural areas of Takum Local Government Area, Taraba State, Nigeria

*Schistosoma ICT IgG-IgM* had the best sensitivity (94.9%) and fairly good specificity (63.9%). *Schistosoma ICT IgG-IgM* had the highest positive predictive value (72.3%), highest negative predictive value (92.6%) as well as highest positive likelihood ratio (2.62). It had the best Youden index (0.58) and highest diagnostic accuracy (0.78) (Table 2).

**Table 2:** Diagnostic performance of tests used for screening urinary schistosomiasis in rural areas of Takum Local Government Area, Taraba State, Nigeria.

Diagnostic tests	Se, %	Sp, %	PPV, %	NPV, %	PLR	YI	DA
	(95% CI)	(95% CI)	(95% CI)	(95% CI)	(95% CI)		
Micro-hematuria	12.9 (0.08 – 18.8)	1.8 (87.1 – 94.9)	61.5 (46.2 – 76.8)	51.3 (46.0 – 56.7)	1.57 (0.87 – 2.92)	0.04	0.54
Proteinuria	54.2 (46.8 – 61.4)	59.3 (52.4 – 66.0)	57.1 (49.8 – 64.4)	56.4 (49.5 – 63.3)	1.33 (1.07 – 1.65)	0.13	0.56
Filtration	35.5 (28.9 – 42.9)	69.0 (62.2 – 75.0)	53.4 (44.7 – 62.2)	51.7 (45.5 – 57.9)	1.14 (0.86 – 1.53)	0.04	0.53
*Schistosoma ICT IgG-IgM*	94.9 (90.4 – 97.4)	63.9 (56.8 – 70.1)	72.4 (66.8 – 78.1)	92.6 (88.1 – 97.0)	2.62 (2.16 – 3.16)	0.58	0.78

Se, sensitivity; Sp, specificity; PPV, positive predictive value; NPV, negative predictive value; PLR, positive likelihood ratio; YI, Youden index; DA, diagnostic accuracy; CI, confidence interval.

## Discussion

The determination of urinary schistosomiasis endemicity in rural areas is very important. *Schistosoma ICT IgG-IgM* had a better positive rate and good concordance with standard WB IgG than the three other techniques used on the field. The least concordance of those techniques exposed the mindful attitude of laboratory technicians misreporting urinary schistosomiasis among infected individuals in rural areas. In this study, *Schistosoma ICT IgG-IgM* became a useful, better and accurate screening test for schistosomiasis. The use of *Schistosoma ICT IgG-IgM* will avoid the misconfidence and untrust of rural inhabitants towards the laboratory examination of schistosomiasis in Primary Health Cares. *Schistosoma ICT IgG-IgM* had a similar report as it was used to screen illegal Africans that resided on sea lands in Italy when they were migrating to various European countries [15], [16]. Proteinuria had the second higher concordance, this could be the effect of *S. haematobium* eggs on the glomeruli leading to excretion of albumin in urine thereby indicating infection with urinary schistosomiasis.

Despite the common use of microhaematuria and proteinuria as reagent strips for screening, previous studies recorded unsatisfactory results [17], [18]. The screening results of microhaematuria and proteinuria varied considerably on the climatic nature of various African areas. In some parts of Africa, the dry climatic condition had higher temperature usually leading to a human dehydration [19]. The higher temperature (36–38 °C) observed in Chanchanji, Manya, Malumshe and Sufa during dry season would have contributed to more concentration of eggs in the bladder thereby causing hematuria and proteinuria. Lower temperatures (24–30 °C) observed in the gallery forest areas (Birama, Barki-Lissa, Kashimbila) led to less inflammation of urine bladder. Inhabitants living in those areas mostly urinate at all times thereby having less concentration of the eggs in the bladder.

Proteinuria had an average sensitivity while microhaematuria had the least. The low and average sensitivities of those tests showed their inability for efficient report of urinary schistosomiasis in endemic areas. Those sensitivities were equally observed in Central Region of Ghana [20]. The higher specificities of microhaematuria and filtration could be due to the immature schistosomes causing less urinary symptoms to infected individuals. *Schistosoma ICT IgG-IgM* had a good sensitivity, fairly good specificity and fairly good positive likelihood ratio. This happened due to good reading of the cassettes used by researchers who assisted the laboratory technicians. The assessment of *Schistosoma ICT IgG-IgM* in Nigeria has been found as a potential and fast screening test for rural endemic areas. The immunology of examined individuals might have also contributed to the false negative and false positive results obtained. An improvement to the development of other immunological new tests is needed to have a very good specificity.

Immunological techniques are better than parasitological techniques used in the laboratory. They determined early schistosomiasis just 14 days after infection [21]. The test effectiveness to determine antibodies of schistosome antigens in infected individuals was better than microscopic observation of the eggs. The antibodies are seriously involved into protective activities of the immune system after schistosomula infection. The Immunoglobulin M (IgM) and Immuglobin G (IgG) are built up immediately in infected individuals to respectively fight schistosomula and immunize the system. The immunoglobulins IgG and IgM on *Schistosoma ICT IgG-IgM* determined schistosomiasis in infected individuals. This guarantees the prescription of Praziquantel drug to treat schistosomiasis in infected individuals. Such individuals will acquire enough protective antibodies for almost two years against future infection [22]. Treatment of schistosomiasis usually engage humoral and cellular immune responses that are mainly for schistosoma-specific antibodies [23]. The various antibodies have several roles in protecting infected individuals against schistosomes. IgG2 and IgG3 have been known as killer of schistosomula in the presence of activated eosonophils [24]. IgG4 was rather suggested as a modulator for the anaphylactic responses associated with IgE [24]. Antibodies such as IgE, IgM and IgG1 have been shown to be resistant to schistosomiasis [25], while IgA was reducing schistosomes fecundity [22], [25]. Chisango et al. [22] reported such benefits of protective immunity after administration of Praziquantel to school children in a chemotherapeutic control of schistosomiasis in Zimbabwe.

*Schistosoma ICT IgG-IgM* had reasonably good positive and high negative predictive values. Eventually, it had a good Cohen’s kappa, fairly good Youden Index and best diagnostic accuracy. *Schistosoma ICT IgG-IgM* was the easiest and most efficient test kit in screening schistosomiasis in endemic areas of sub-Saharan Africa.

Economically, *Schistosoma ICT IgG-IgM* was expensive for sub-Saharan African countries. The financial cost needs to be curtailed by the company for those countries. This will help such countries to acquire the test kits for fast screening. It will equally help to implement treatment to ill individuals in endemic areas. Most rural inhabitants in sub-Saharan Africa are poor and could not afford such test kits at that expensive amount. The poverty of such population is ineffable. The various companies around the world should manufacture and sell at affordable price.

The first limitation of *Schistosoma ICT IgG-IgM* was the preparation of plasma or serum*.* Inhabitants disagreed with the test as they observed the laboratory technicians collecting blood for plasma or serum. The rural inhabitants had contrary minds because of their indigenous practices. Secondly, the immune-chromatography technology test kit was expensive.

## Conclusions

*Schistosoma ICT IgG-IgM* was a suitable and good diagnosis test in screening urinary schistosomiasis in sub-Saharan Africa. *Schistosoma ICT IgG-IgM* had the best sensitivity, fairly good specificity and fairly good positive likelihood ratio. The test kit had a fairly good Youden index and better diagnosis accuracy. It is recommended that the manufacturer LDBIO Diagnostics, Lyon, France and other companies develop similar tests that could use blood directly instead of serum.

## Supplementary Material

Supplementary Material
