# Supplementary figures and images for: Diagnostic accuracy of Schistoso ma ICT Ig G - IgM and comparison to other used techniques screening urinary schistosomiasis in Nigeria
Source: Adv Lab Med. 2021 Feb 9;2(1):71–7. doi: 10.1515/almed-2020-0093 (PMC10197290; doi:10.1515/almed-2020-0093)

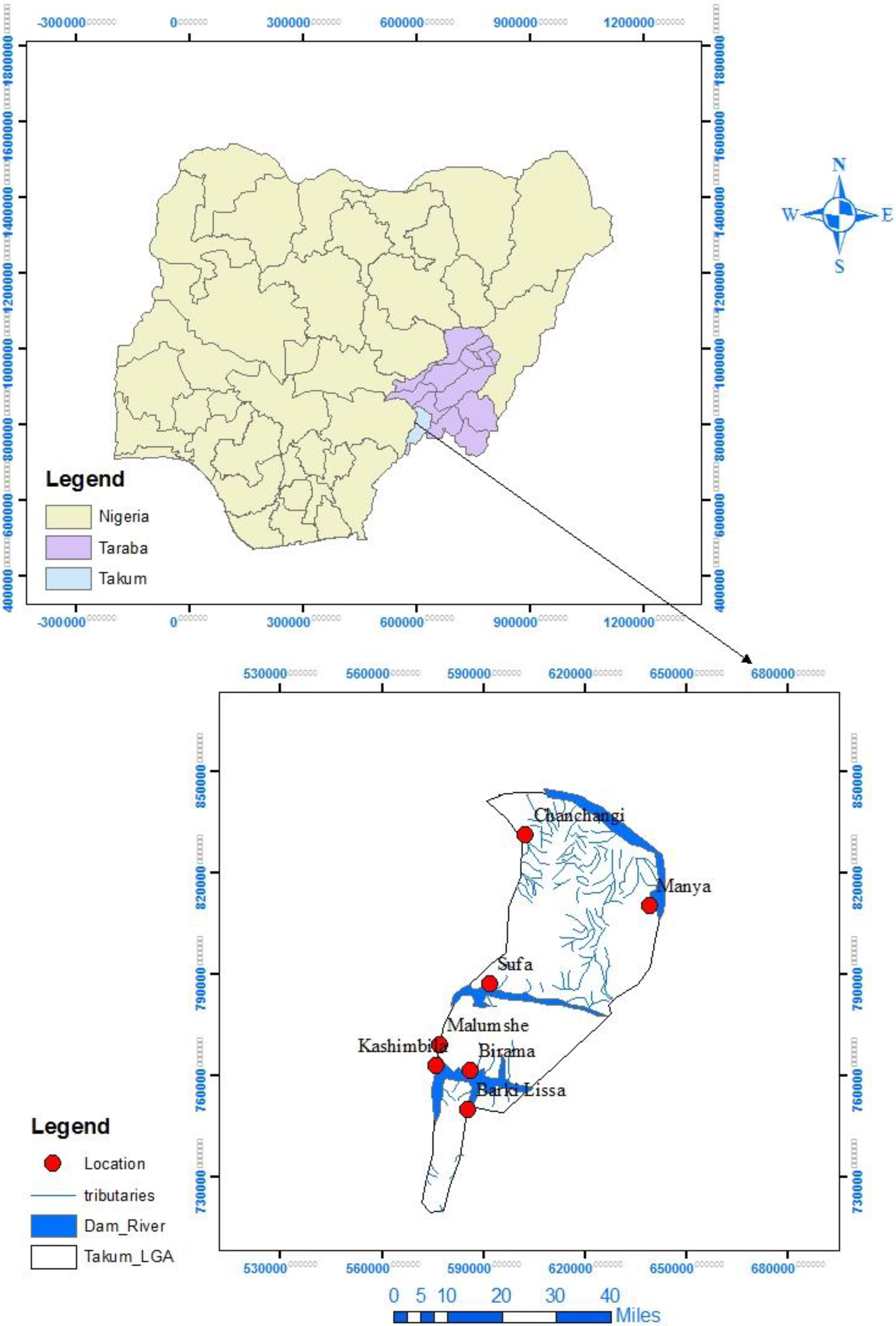

Supplement: Supplementary file 1 — Supplementary Material [file j_almed-2020-0093_suppl.zip › j_almed-2020-0093_suppl.tif]
